# Biological control of bacterial plant diseases with *Lactobacillus plantarum* strains selected for their broad‐spectrum activity

**DOI:** 10.1111/aab.12476

**Published:** 2018-11-26

**Authors:** Núria Daranas, Gemma Roselló, Jordi Cabrefiga, Irene Donati, Jesús Francés, Esther Badosa, Francesco Spinelli, Emilio Montesinos, Anna Bonaterra

**Affiliations:** ^1^ Institute of Food and Agricultural Technology‐CIDSAV‐XaRTA University of Girona Girona Spain; ^2^ Department of Agricultural and Food Sciences (DISTAL), Alma Mater Studiorum University of Bologna Bologna Italy

**Keywords:** bacterial plant diseases, *Lactobacillus plantarum*, *Pseudomonas syringae* pv. *actinidiae*, *Xanthomonas arboricola* pv. *pruni*, *Xanthomonas fragariae*

## Abstract

The use of lactic acid bacteria (LAB) to control multiple pathogens that affect different crops was studied, namely, *Pseudomonas syringae* pv. *actinidiae* in kiwifruit, *Xanthomonas arboricola* pv. *pruni* in *Prunus* and *Xanthomonas fragariae* in strawberry. A screening procedure based on in vitro and *in planta* assays of the three bacterial pathogens was successful in selecting potential LAB strains as biological control agents. The antagonistic activity of 55 strains was first tested in vitro and the strains *Lactobacillus plantarum* CC100, PM411 and TC92, and *Leuconostoc mesenteroides* CM160 and CM209 were selected because of their broad‐spectrum activity. The biocontrol efficacy of the selected strains was assessed using a multiple‐pathosystem approach in greenhouse conditions. L. plantarum PM411 and TC92 prevented all three pathogens from infecting their corresponding plant hosts. In addition, the biocontrol performance of PM411 and TC92 was comparable to the reference products (Bacillus amyloliquefaciens D747, *Bacillus subtilis* QST713, chitosan, acibenzolar‐S‐methyl, copper and kasugamycin) in semi‐field and field experiments. The in vitro inhibitory mechanism of PM411 and TC92 is based, at least in part, on a pH lowering effect and the production of lactic acid. Moreover, both strains showed similar survival rates on leaf surfaces. PM411 and TC92 can easily be distinguished because of their different multilocus sequence typing and random amplified polymorphic DNA profiles.

## 1 INTRODUCTION

Increased global trade, together with climate change and the limitations in plant protection products, has favoured the emergence and establishment of new plant diseases which, in turn, cause significant crop losses (Lamichhane et al., 2015; Yáñez‐López et al., 2012). Fruit production, for instance, is threatened by several bacterial plant diseases such as the bacterial canker of kiwifruit caused by *Pseudomonas syringae* pv. *actinidiae* (Psa), the bacterial spot of stone fruits caused by *Xanthomonas arboricola* pv. *pruni* (Xap) and the angular leaf spot of strawberry caused by *Xanthomonas fragariae* (Xf) (Donati et al., 2014; Kim et al., 2016; Lamichhane, 2014). The European and Mediterranean Plant Protection Organization (EPPO) lists Psa, Xap and Xf as quarantine organisms.

Managing these diseases mainly relies on preventive applications of bactericides containing copper compounds or antibiotics (Cameron & Sarojini, 2014; Lamichhane, 2014). However, the selection of resistant pathogen populations and phytotoxicity are the main drawbacks to this practice (Lalancette & McFarland, 2007; McManus, Stockwell, Sundin, & Jones, 2002). Overall, reliance on conventional pesticides needs to be reduced and an integrated pest management (IPM) framework implemented (Lamichhane et al., 2015). The plant defence elicitor acibenzolar‐S‐methyl (ASM) has been reported as being a potential alternative compound for managing bacterial canker of kiwifruit (Cellini et al., 2014) and angular leaf spot of strawberry (Braun & Hildebrand, 2013). Likewise, chitosan exhibits antimicrobial activity and acts as an elicitor of plant defence mechanisms, making it a potential alternative agent for managing bacterial canker of kiwifruit (Cameron & Sarojini, 2014). Nevertheless, phytotoxicity and a high variability in the response in host plants in the field have been reported, which raises questions about their feasibility in crop protection (Reglinski et al., 2013). Therefore, interest in selecting beneficial microorganisms with which to develop biological control agents (BCAs) has increased as a result of microbial biopesticides being an indispensable and powerful tool in IPM (Matyjaszczyk, 2015). While strains of bacteria, fungi and viruses to manage plant diseases and pests are now commercially available (Matyjaszczyk, 2015; Montesinos & Bonaterra, 2017), because of the influence biotic and abiotic factors have, the efficacy of the biological products may vary between trials or decrease in field conditions (Sundin, Werner, Yoder, & Aldwinckle, 2009). Such limitations have stimulated the search for novel strains of microorganisms that have a broad spectrum of antagonistic activity against plant pathogens. Lactic acid bacteria (LAB) are an interesting group often found in a plant‐associated microbiome (Trias, Bañeras, Badosa, & Montesinos, 2008; Zwielehner et al., 2008).

LAB are good candidates to develop microbial biopesticides with, because they include some strains which have been categorised by the U.S. Food and Drug Administration as Generally Regarded as Safe and by the European Food Safety Authority as having Qualified Presumption of Safety. Furthermore, many LAB strains show antimicrobial activity thanks to the production of active metabolites such as organic acids, bacteriocins and several inhibitory bioactive compounds (Reis, Paula, Casarotti, & Penna, 2012). LAB have been widely reported as being biopreservatives of vegetables and fruits, inhibiting the growth of foodborne bacterial pathogens and spoilage fungi (Crowley, Mahony, & Van Sinderen, 2013; Trias, Bañeras, Badosa, & Montesinos, 2008; Trias, Bañeras, Montesinos, & Badosa, 2008). In addition, some LAB strains have also been reported as being potential BCA against several bacterial plant pathogens (Roselló et al., 2013; Tsuda et al., 2016; Visser, Holzapfel, Bezuidenhout, & Kotze, 1986).

BCA must be carefully selected because not all species or strains confer plant protection against pathogens. Screening strategies enabling the selection of strains with pathogen suppressive activity include in vitro antagonism tests and the assessment of infection prevention in detached plant organs and whole plants (Haidar et al., 2016; Köhl, Postma, Nicot, Ruocco, & Blum, 2011; Roselló et al., 2013). Moreover, keen commercial interest in LAB has fuelled studies to typify the most promising strains, as their identification and characterisation is a requirement for BCA registration. Typing techniques are based on DNA analysis and two of the most commonly used are multilocus sequence typing (MLST) (de las Rivas et al., 2006; Tanganurat et al., 2009) and random amplified polymorphic DNA‐PCR (RAPD‐PCR) (López, Torres, & Ruiz‐Larrea, 2008).

The aims of the present study were fourfold: (a) screen plant‐associated LAB using in vitro tests and select antagonistic strains with broad‐spectrum activity against Psa, Xap and Xf, (b) assess the biocontrol efficacy of the selected strains in preventing infections by the three pathogens in potted plants (kiwifruit, *Prunus* and strawberry) in the greenhouse, (c) compare the biocontrol performance of the selected strains to reference products in semi‐field and field experiments and (d) characterise the selected strains in regards to the mechanisms involved in the in vitro antibacterial activity against Psa, Xap and Xf and MLST and RAPD‐PCR profiling.

## 2 MATERIALS AND METHODS

### 2.1 Bacterial strains and culture conditions

A total of 55 plant‐associated LAB isolates from the culture collection of the Institute of Food and Agricultural Technology and Center for Innovation and Development of Plant Health (INTEA‐CIDSAV) were selected for this study (Table 1). Spontaneous rifampicin resistant mutants of wild‐type *Lactobacillus plantarum* PM411 and TC92 (PM411R and TC92R) were used in the plant colonisation studies (Roselló et al., 2013).

**Table 1:** Lactic acid bacteria and bacterial plant pathogen strains used in this study

Species	Code strain^a^	Host	Geographical origin	Growth medium^b^
Lactic acid bacteria^c^				
*Lactobacillus plantarum*	AC58, AC59, AC73, AC81, AC84	Aubergine	Spain	MRS
	BC24, BC30, BC37, BC50, BC66	Chard	Spain	MRS
	CC31, CC100, CC121	Cucumber	Spain	MRS
	CC70, CC85, CC93	Cabbage	Spain	MRS
	CM450	Courgette	Spain	MRS
	CM466	Persimmon	Spain	MRS
	FC248, FC534	Fig	Spain	MRS
	NC568	Loquat	Spain	MRS
	PC40, PC49, PC67	Potato	Spain	MRS
	PM314, PM340, PM411, PM411R^d^	Pear	Spain	MRS
	RC526	Blackberry	Spain	MRS
	TC26, TC28, TC35, TC41, TC43, TC44, TC46, TC54, TC60, TC69, TC71, TC92, TC92R^c^, TC97, TC101, TC102, TC110, TM106	Tomato	Spain	MRS
*Lactobacillus pentosus*	SE217, SE294, SE304, SE307	Soya beans	Spain	MRS
	BM305	Broccoli	Spain	MRS
*Leuconostoc mesenteroides*	CM160	Cherry	Spain	MRS
	CM209	Lettuce	Spain	MRS
	PM366	Peach	Spain	MRS
*Lactococcus lactis*	SE303	Soya beans	Spain	MRS
Non‐identified	FC560	Fig	Spain	MRS
Bacterial plant pathogens				
Psa	CFBP7286, CFBP7286‐GFPuv^e^	*Actinidia chinensis*	Italy	Luria‐Bertani
	NCPPB3739	*Actinidia deliciosa*	Japan	Luria‐Bertani
	IVIA 3700‐1^f^	*A. deliciosa*	Portugal	Luria‐Bertani
Xap	CFBP3894	*Prunus salicina*	New Zealand	Luria‐Bertani
	CFBP5563	*Prunus persica*	France	Luria‐Bertani
Xf	IVIA XF349‐9A^f^	*Fragaria vesca*	Spain	B medium
	CECT549	*Fragaria chiloensis var. ananassa*	USA	B medium

CECT: Colección Española de Cultivos Tipo; CFBP: La Collection Française de Bactéries Phytopathogènes; INTEA‐CIDSAV: Institute of Food and Agricultural Technology and Center for Innovation and Development of Plant Health; IVIA: Instituto Valenciano de Investigaciones Agrarias; LAB: lactic acid bacteria; NCPPB: National Collection of Plant Pathogenic Bacteria; Psa: *Pseudomonas syringae* pv. *actinidiae*; Xap, *Xanthomonas arboricola* pv. *pruni*; Xf, *Xanthomonas fragariae*.

a CFBP (Institut National de la Recherche Agronomique (INRA), France); CECT (Valencia, Spain); IVIA (Valencia, Spain); NCPPB (Fera, UK).

b MRS (de Man, Rogosa and Sharpe), B medium (Hazel & Civerolo, 1980).

c INTEA‐CIDSAV culture collection (Roselló et al., 2013; Trias, Bañeras, Badosa, & Montesinos, 2008; Trias, Bañeras, Montesinos, & Badosa, 2008). LAB strains were identified at species level based on 16S rDNA sequences. *L. plantarum* “group” was confirmed using species‐specific primers (PLANT1/LOWLAC) (Chagnaud, Machinis, Coutte, Marecat, & Mercenier, 2001) by PCR amplification. Positive isolates for species‐specific PCR were then tested by multiplex PCR in a second step for the identification of *L. plantarum*, *L. paraplantarum* and *L. pentosus* with *recA* gene‐based primers paraF, pentF, planF and pREV (Torriani, Felis, & Dellaglio, 2001).

d Spontaneous mutants of *L. plantarum* PM411 and TC92 strains resistant to rifampicin.

e Courtesy of Dr. F. Spinelli, Department of Agricultural and Food Sciences, University of Bologna, Italy (Spinelli, Donati, Vanneste, Costa, & Costa, 2011).

f Courtesy of Dra M. M. Lopez, IVIA, Valencia, Spain.

Three strains of *P. syringae* pv. *actinidiae* (Psa) and two strains of Xap and Xf were used as target bacteria in the in vitro antagonism tests and biocontrol efficacy assays (Table 1). Psa CFBP7286‐GFPuv, which is resistant to kanamycin, was used for the colonisation studies in kiwifruit plants (Spinelli et al., 2011).

Cultures were prepared for routine use from the isolates preserved at −80°C. LAB were grown on de Man, Rogosa and Sharpe (MRS) agar (Oxoid, Basingstoke, UK) at 23°C for 48 h. Psa and Xap were grown on Luria‐Bertani agar at 23°C for 24 hr and Xf was grown on B medium (Hazel & Civerolo, 1980) at 23°C for 24 hr. Bacterial suspensions were prepared in distilled water at 1‐5 x 10^8^ colony forming units (CFU)/mL.

### 2.2 Plant material and greenhouse conditions

Two‐year‐old kiwifruit plants and 15‐ to 30‐day‐old kiwifruit plantlets (*Actinidia chinensis* var. *deliciosa* cv. Hayward), 15‐ to 30‐day‐old *Prunus amygdalus* × *Prunus persica* plantlets (cv. GF‐677), and cold‐stored strawberry plants (*Fragaria* × *ananassa* cv. Darselect) were obtained from commercial nurseries (SolJardí, Jafre, Spain; Vitroplant, Cesena, Italy; Agromillora Iberica, Barcelona, Spain; and Planasa, Valtierra, Spain, respectively). Potted plants with about 8 to 10 leaves per plant were used and were kept in a greenhouse at 26 ± 2°C, 60 ± 10% relative humidity (RH) and a 16:8 hr light:dark photoperiod. Standard nitrogen‐phosphate‐potassium (NPK) fertilisation and irrigation, as well as insecticide and miticide sprays were applied. Plants inoculated with Psa, Xap or Xf were maintained in a class II greenhouse (i.e., an EPPO A2 level quarantine biosafety greenhouse).

### 2.3 In vitro antagonistic activity

The antagonistic activity of 55 LAB isolates was assayed in vitro on Psa (NCPPB3739 and IVIA 3700‐1), Xap (CFBP3894 and CFBP5563) and Xf (IVIA XF349‐9A and CECT549). The experiment was performed twice using two procedures. One assay was carried out with the agar spot test in lactose‐bromocresol purple agar (LBP) as in Trias, Bañeras, Badosa, and Montesinos (2008), albeit with some modifications. Specifically, LBP soft agar (0.7% agar) was mixed with Psa or Xap suspension, while B medium soft agar was mixed with Xf suspension. For the other assay, 5‐mm‐in‐diameter discs cut from 24‐h‐old cultures of LAB isolates on MRS agar were deposited on the surface of plates containing cultures of the target pathogen. 0.5 mL of the target pathogen suspension at 5 × 10^7^ CFU/mL was mixed in 4.5 mL of Luria‐Bertani (Psa and Xap) or with B medium (Xf) soft agar (0.7% agar) and overlaid on the plate containing the same media. Plates were incubated at 23°C, and the diameter of the zone of inhibition was measured after 24 and 48 hr.

### 2.4 Biocontrol efficacy assays under greenhouse conditions

A total of five LAB isolates (*L. plantarum* CC100, PM411, TC92 and *Leuconostoc mesenteroides* CM160 and CM209) were evaluated. Potted two‐year‐old kiwifruit plants, *Prunus*, and strawberry plants were sprayed to runoff with a 10^8^ CFU/mL suspension of LAB cells using a hand‐sprayer (Herkules, Nuair, Robassomero, Italy). The plants were then kept in plastic bags in the greenhouse in order to reach high RH conditions. After 24 hr, plants were inoculated with a suspension of the corresponding pathogen at 1–5 × 10^8^ CFU/mL (IVIA 3700‐1 of Psa, CFBP5563 of Xap or CECT549 of Xf). The pathogen suspensions were mixed with diatomaceous earth (1 mg/mL) and applied to runoff using a hand‐sprayer. Once again, the plants were covered with plastic bags for 24 hr and kept in a class II greenhouse. Streptomycin‐treated (Sigma, St. Louis, MO) (100 mg/L) and water‐treated plants were included as controls. Disease incidence was calculated as the percentage of infected leaves and was determined in each replicate at 15 to 21 days post inoculation.

### 2.5 Survival of L. plantarum PM411 and TC92 on leaves

The potted plants were sprayed to runoff with a 10^8^ CFU/mL cell suspension of PM411R (in the case of strawberry and 15‐ to 30‐day‐old kiwifruit plantlets) or TC92R (*Prunus*), then covered with plastic bags and maintained in the greenhouse as described above. The monitoring of PM411R and TC92R population levels was performed as Roselló, Francés, Daranas, Montesinos, and Bonaterra (2017) described. Three leaves were taken from each replicate at 0, 1, 2, 5, 8 and 10 days post inoculation. The population levels of PM411R and TC92R were expressed as Log_10_ CFU per leaf.

### 2.6 Effect of L. plantarum PM411 on Psa survival in kiwifruit plants

Potted kiwifruit plants (15‐ to 30‐day‐old plantlets) were sprayed with a 10^8^ CFU/mL cell suspension of PM411, covered with plastic bags and maintained in the greenhouse as described above. After 24 hr, the plants were inoculated with Psa suspension at 1–5 × 10^8^ CFU/mL (CFBP7286‐GFPuv) to runoff using a hand‐sprayer, covered with plastic bags and maintained in the greenhouse. Streptomycin‐treated (Sigma) (100 mg/L) and water‐treated plants were included as controls. To monitor epiphytic and endophytic Psa populations, three leaves per replicate were sampled at 1 and 4 days post inoculation. Leaves were weighed and vigorously homogenised in 20 mL of 50‐mM sterile phosphate buffer and 0.1% peptone for 5 min to collect the epiphytic population. The same leaves were also used to assess the endophytic population consistent with Cellini et al. (2018). Appropriate dilutions of epiphytic and endophytic samples were seeded onto Luria‐Bertani agar plates amended with 100 μg/mL of kanamycin (Sigma) to select CFBP7286‐GFPuv and 100 μg/mL of cycloheximide (Sigma) to avoid fungal growth. Plates were incubated at 23°C for 48 hr, and the green fluorescent colonies were counted under UV light. The epiphytic and endophytic population levels of Psa were expressed as Log_10_ CFU per leaf or g, respectively.

### 2.7 Biocontrol efficacy assays under semi‐field conditions

The efficacy of *L. plantarum* PM411 and TC92 in controlling Psa, Xap and Xf was studied in semi‐field assays and compared to reference products. The semi‐field assays consisted of treatment applications in the field and keeping plants there for 7 days before being transported to a class II greenhouse for pathogen inoculation.

Potted two‐year‐old kiwifruit plants were taken to an experimental orchard located in Zevio (Verona, Italy). The treatments administered were: (a) PM411 cell suspension at 10^8^ CFU/mL prepared as described above, (b) *Bacillus amyloliquefaciens* D747 (Amylo‐X, 25% w/w a.i., 5 × 10^10^ CFU/g; Biogard, Monza Brianza, Italy) at 0.375 g a.i. L^−1^, (c) copper oxide (Nordox, 75% w/w a.i.; Comercial Química Massó, Barcelona, Spain) at 0.45 g a.i. L^−1^.

Potted *Prunus* and strawberry plants were taken to the experimental orchard located at the Mas Badia Agricultural Experiment Station (Girona, Spain). The treatments performed were: (a) TC92 or PM411 cell suspension at 10^8^ CFU/mL prepared as described above, (b) *Bacillus subtilis* QST713 (Serenade Max, 15.67% w/w a.i.; Bayer Crop Science, Monheim am Rhein, Germany) at 0.55 g a.i. L^−1^, (c) chitosan (Biorend, 2.5% v/v a.i.; Bioagro, Santiago, Chile) at 7.5 g a.i. L^−1^, (d) ASM (Bion, 50% w/w a.i.; Syngenta, Basel, Switzerland) at 0.075 g a.i. L^−1^, (e) copper hydroxide (Kocide, 35% w/w a.i; Certis, Elche, Spain) at 1.05 g a.i. L^−1^ and (f) kasugamycin (Kasumin, 8% w/w a.i; Lainco, Barcelona, Spain) at 0.16 g/L. In all the experiments, water‐treated plants were included as controls. All the treatments, except for kasugamycin, were applied twice, 7 and 1 days before inoculation, using a hand‐sprayer to runoff.

Plants were spray‐inoculated with the corresponding pathogen suspension at 10^8^ CFU/mL (Psa CFBP7286, Xap CFBP5563, Xf CECT549) as described in the greenhouse experiments. After inoculation, plants were covered with plastic bags for 48 hr and maintained in the class II greenhouse. The disease incidence was determined as described earlier (see greenhouse experiments) for each replicate at 15–21 days post inoculation.

### 2.8 Biocontrol efficacy assays in orchard conditions

The field experiment was carried out in 2017 at a commercial orchard (*A. chinensis* var. *deliciosa*, cv. Hayward) located in Sarna, close to Faenza (Emilia Romagna, Italy). The disease had been present in this orchard in previous years with a moderate pressure. Standard cultural management (i.e., fertigation, green and winter pruning, thinning and assisted pollination) was adopted. For each plant (experimental design explained below), four shoots without Psa symptoms were selected and tagged at the beginning of the experiment. The treatments performed were: (a) PM411 cell suspension at 10^8^ CFU/mL prepared as described above, (b) *B. amyloliquefaciens* D747 (Amylo‐X 5 × 10^10^ CFU/g) at 0.375 g a.i. L^−1^, and (c) copper oxide (Nordox) at 0.45 g a.i. L^−1^. Water‐treated plants were included as controls. All the treatments were applied every 14 days or after each rainfall (≥4 mm of rain), in the case the rain event occurred at 7 or more days after the treatment. Copper applications started at bud break (phenological Biologische Bundesanstalt, Bundessortenamt und CHemische Industrie (BBCH) 03) and were repeated until fruit grew to 30% of the final size (BBCH 73). BCA applications were performed at 10, 50 and 100% of blooming and were repeated until BBCH 73 was reached. Psa incidence was assessed twice during the season, with the second assessment taking place once disease progression had been halted and was calculated as the percentage of symptomatic leaves on 20 shoots per repetition. Psa symptomatology was confirmed by molecular identification following Gallelli, Talocci, L'aurora, and Loreti (2011).

### 2.9 Characterising PM411 and TC92 strains

The role the different metabolites played on the antibacterial activity of PM411 and TC92 strains against target pathogens was studied together with a genotypic characterisation.

#### 2.9.1 Metabolite profiling

Agar diffusion assays using cell‐free supernatants (CFSs) were performed. PM411 and TC92 strains were grown in MRS broth for 24 hr at 30°C with shaking (100 rpm). CFS were obtained by centrifugation (10,000 *g* for 10 min) (5,810 R; Eppendorf, Hamburg, Germany) and were filtered (Whatman FP30/0.45; Millipore, Bedford, MA). 20 μL of CFS was deposited on the surface of plates containing cultures of the target pathogens (Psa IVIA 3700‐1, Xap CFBP3894, and Xf IVIA XF349‐9A) prepared as described above (see in vitro antagonism tests, Luria‐Bertani for Psa and Xap and B medium for Xf). Plates were incubated at 23°C, and the diameter of zone of inhibition was examined at 24 and 48 hr. Fractions of CFS were exposed to different treatments (neutralised CFS, and neutralised CFS treated with proteinase K, trypsin, α‐chymotrypsin or catalase) as described by Trias, Bañeras, Montesinos, and Badosa (2008), and the antimicrobial activity was assessed by agar diffusion assay (as described above). Three independent replicates for each CFS fraction were performed. Lactic acid was quantified in CFS using an Enzytec D‐/L‐Lactic Acid commercial kit (Boehringer Mannheim/R‐Biopharm AG, Darmstadt, Germany) following the manufacturer's instructions. 1:10 diluted CFS with redistilled water was used for the measurement. Two experiments were carried out with three independent replicates.

#### 2.9.2 Molecular characterisation of PM411 and TC92

1. DNA extraction. Genomic DNA from cell suspensions at 10^8^ CFU/mL of the 45 *L. plantarum* isolates (INTEA‐CIDSAV culture collection, Table 1) was extracted as described by Llop, Caruso, Cubero, Morente, and López (1999). The concentration and purity of the DNA was assessed using a NanoDrop ND‐1000 Spectrophotometer (Thermo Fisher Scientific, Waltham, MA).
2. PCR amplifications and conditions. Amplification mixtures and PCR conditions for MLST and RAPD‐PCR analysis are described in Table 2. The amplification products were separated by electrophoresis on a 1.5% (w/v) agarose gel in 1× Tris‐acetate Disodium ethylenediaminetetraacetate dihydrate (EDTA) and stained with Sybr Safe (SYBR Safe, Invitrogen, Life Technologies, Carlsbad, CA). Gel images were captured with an imaging system (FX‐20M; Vilvert, Lourmat, France).
3. MLST analysis. The housekeeping genes encoding the following proteins were chosen for analysis: phosphoglucomutase (*pgm*), D‐alanine‐D‐alanine ligase (*ddl*), B subunit of DNA gyrase (*gyrB*), ATPase subunit of the phosphoribosylaminoimidazole carboxylase (*purK1*), glutamate dehydrogenase (*gdh*) and DNA mismatch repair protein (*mutS*). The amplification and sequencing primers used have been previously described by de las Rivas et al. (2006), except for the *gdh* gene where the primers were designed in this work (gdhF 5′‐GGTTACACCCATCCGTTAAT‐3′ and gdhR 5′‐TTCTTCAAAAGTCCAGTCA‐3′, 901‐bp fragment). Amplification products were purified with a desalting and concentrating DNA solutions kit QIAEX (QIAGEN GmbH, Hilden, Germany) for direct sequencing using the Automatic Sequencer 3730XL (Macrogen, Seoul, South Korea). Sequence alignments and comparisons were performed using a BioEdit Sequencing Editor (http://www.mbio.ncsu.edu/bioedit/bioedit.html). For each gene, the sequences obtained in this study from the 45 *L. plantarum* isolates (Table 1) were compared to each other and to the sequences of 26 *L. plantarum* isolates previously reported (de las Rivas et al., 2006; Tanganurat et al., 2009). Allele numbers were assigned following the codes previously described (de las Rivas et al., 2006; Tanganurat et al., 2009). New allele sequences were deposited in the GenBank database under the accession numbers KT247498 (allele 4 *pgm*), KT247499 (allele 7 *ddl*), KT247496 (allele 9 *purK1*), KT247497 (allele 10 *purK1*), KT247501 (allele 11 *gdh*) and KT247500 (allele 9 *mutS*). Each strain was defined by an allele profile or sequence type (ST) as described in de las Rivas et al. (2006).
4. RAPD‐PCR analysis. PCR amplification of the 45 *L. plantarum* isolates was carried out. The primers P3 (Tailliez, Tremblay, Dusko Ehrlich, & Chopin, 1998), Inva1 (Rahn et al., 1992), 512Fb (Holt & Cote, 1998), P4 and P7 (Di Cagno et al., 2010), with arbitrarily chosen sequences, were used singly in separate reactions. Pattern analyses were performed with Image Lab version 4.1 software (Bio‐Rad Laboratories, Hercules, CA).

**Table 2:** Amplification mixtures and PCR conditions

PCR approach	Amplification mixture^a^	PCR conditions^b^
MLST	1× PCR buffer, 2.5‐mM MgCl_2_, 0.2‐mM dNTPs, 0.2 μM each primer, 1 U *Taq* and 20‐ng DNA (reaction vol, 25 μL)	95°C for 10 min; 30 cycles of 95°C for 30 s, 54°C (*gdh*) or 48°C (*pgm, ddl, gyrB, purK1, mutS*) for 60 s, 72°C for 60 s; and elongation 72°C for 10 min.
RAPD‐PCR	1× PCR buffer, 1.5‐mM MgCl_2_, 0.2‐mM dNTPs, 0.2 μM each primer, 3.75 U *Taq*, and 50‐ng DNA (reaction vol, 25 μL)	For P3 and P4: 94°C for 3 min; 30 cycles of 94°C for 1 min, 36°C for 2 min, 72°C for 2 min; and elongation at 72°C for 2 min. For 512Fb, Inva1 and P7: 94°C for 4 min; 45 cycles of 94°C for 1 min, 35°C for 1 min, 72°C for 1 min; and elongation at 72°C for 5 min.

dNTPs: deoxynucleotides; MLST: multilocus sequence typing; RAPD‐PCR: random amplified polymorphic DNA‐PCR.

a dNTPs (Invitrogen Life Technologies, Carlsbad, CA); primers (Sigma Aldrich, Barcelona, Spain); *Taq*, Taq DNA polymerase (Biotools, Madrid, Spain for MLST and Invitrogen for RAPD‐PCR).

b PCR was carried out in a GeneAmp PCR system 9,700 (Applied Biosystems).

### 2.10 Experimental designs and data analysis

In vitro antagonism tests consisted of three independent replicates of each LAB and were performed twice. The diameters of the zones of inhibition were transformed into binary data (“1” for presence and “0” for absence of inhibition zone) for hierarchical cluster analysis. The simple matching coefficient of similarity using the unweighted pair group method with the arithmetic average (UPGMA) as the cluster algorithm was used (Numerical Taxonomy System program package NTSYSpc, Exeter Software, New York City, NY). For each target pathogen strain, a value of “0” in both assays was considered as no inhibition activity, a value of “1” in only one assay was considered as moderate inhibition activity, while a value of “1” in both assays was considered as high inhibition activity.

Biocontrol efficacy assays in greenhouse and semi‐field conditions consisted of three independent biological replicates per treatment with five plants per replicate. Two independent experiments were performed in all three pathosystems in the greenhouse and semi‐field experiments except for the semi‐field and monitoring assays in Psa that were performed once. The biocontrol efficacy assay for Psa in the field consisted of four randomised blocks of five plants per treatment. Each block was repeated in separated rows. Disease incidence and Psa population levels were subjected to analysis of variance, and mean values were compared using the least significant difference test at *p* < .05. The analysis was performed with the General Linear Model (GLM) procedure of the Statistical Analysis System for Personal Computers (PC‐SAS) (Version 8.2; SAS Institute Inc., Cary, NC).

The monitoring of the LAB populations consisted of three independent biological replicates with five plants per replicate and two independent experiments.

The allelic profile of each *L. plantarum* strain obtained with MLST analysis was used to investigate clonal complexes (CCs) using the minimum spanning tree (MST) method (Bionumerics v7.5, Applied‐Maths, Sint‐Martens‐Latem, Belgium). Polymorphic DNA bands obtained with RAPD analysis were transformed into a binomial matrix (“1” for presence and “0” for absence of fragments). A dendrogram with the five series of RAPD‐PCR profiles of the 45 *L. plantarum* isolates was generated using the Dice coefficient of similarity and the UPGMA method (NTSYSpc).

## 3 RESULTS

### 3.1 Antagonistic activity against bacterial plant pathogens

**Figure 1:**
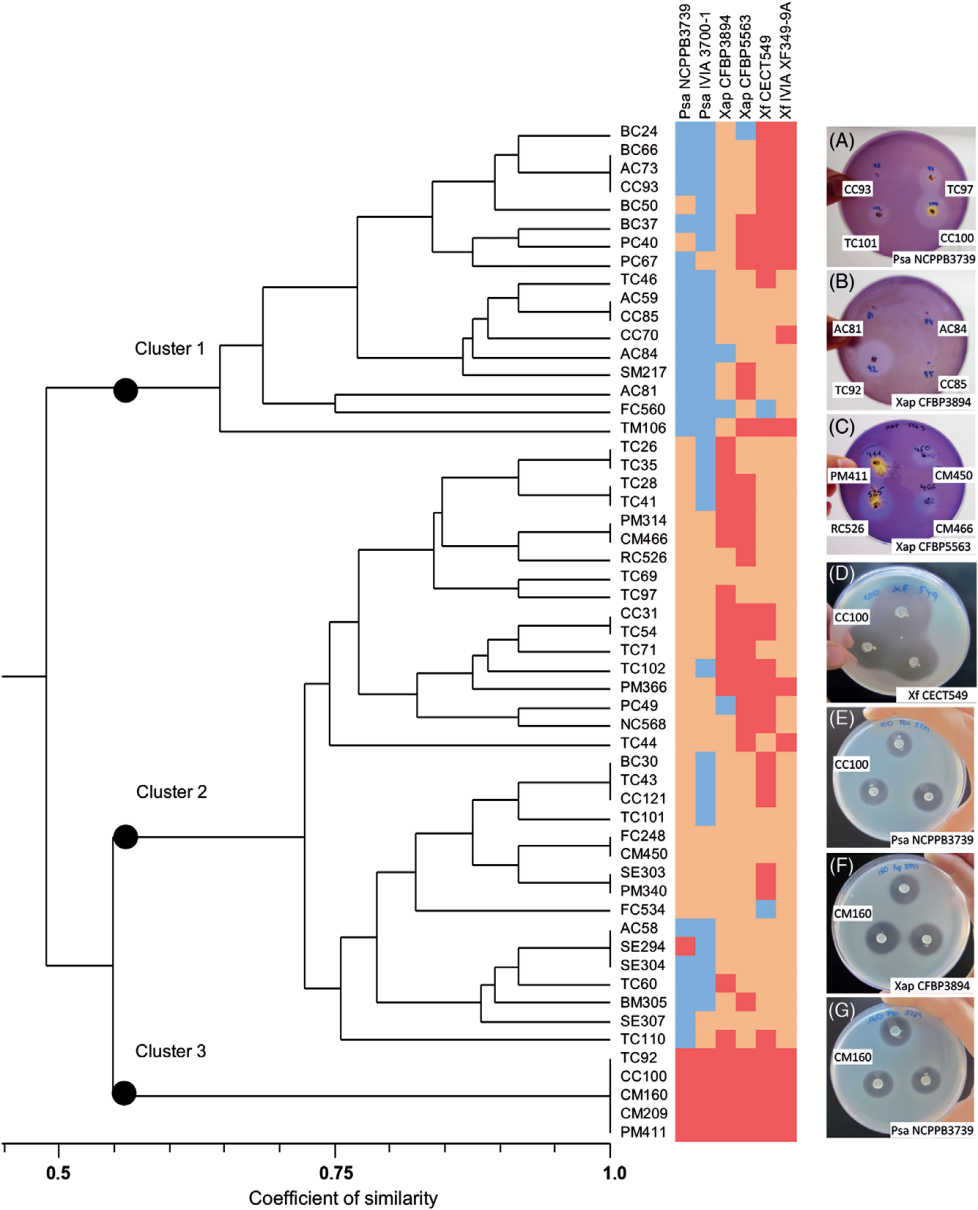
Dendrogram of the in vitro antagonism spectrum of 55 lactic acid bacteria (LAB) strains. The target pathogens are listed at the top and correspond to strains NCPPB3739 and IVIA 3700‐1 of Psa, strains CFBP3894 and CFBP5563 of Xap and strains CECT549 and IVIA XF349‐9A of *XF*. Two independent experiments were performed. Clusters of strains are indicated by a dot. Colour legend indicates the inhibition activity: Pale grey, negative in both experiments; dark grey, positive in one experiment; black, positive in both experiments. Cluster analysis was performed using the UPGMA and with the simple matching coefficient of similarity. The photographs A, B, and C show the antimicrobial activity obtained by the agar spot test in LBP, and the photographs D, E, F and G show the antimicrobial activity obtained by the disc test. IVIA, Instituto Valenciano de Investigaciones Agrarias; Psa: *Pseudomonas syringae* pv. *actinidiae*; Xap, *Xanthomonas arboricola* pv. *pruni*; UPGMA, unweighted pair group method with the arithmetic average; *XF*, *Xanthomonas fragariae*

Several LAB isolates severely inhibited growth (inhibition zone diameter > 10 mm) of Psa (NCPPB3739 and IVIA 3700‐1), Xap (CFBP3894 and CFBP5563) and Xf (IVIA XF349‐9A and CECT549). However, differences in sensitivity among Psa, Xap and Xf towards LAB were observed (see photographs Figure 1). The cluster analysis showed three main groups of antagonism spectrum at a similar level of 0.55 (Figure 1). Cluster 1 included 17 strains that generally showed no activity against Psa, moderate activity against Xap and moderate or high activity against Xf. Cluster 2 included 33 strains that generally showed no or moderate activity against Psa, and moderate or high activity against Xap and Xf. Interestingly, Cluster 3 included five strains—CC100, CM160, CM209, PM411 and TC92—that displayed broad and generally high activity against all the target pathogens.

### 3.2 Psa, Xap and Xf control in plants under greenhouse conditions

**Figure 2:**
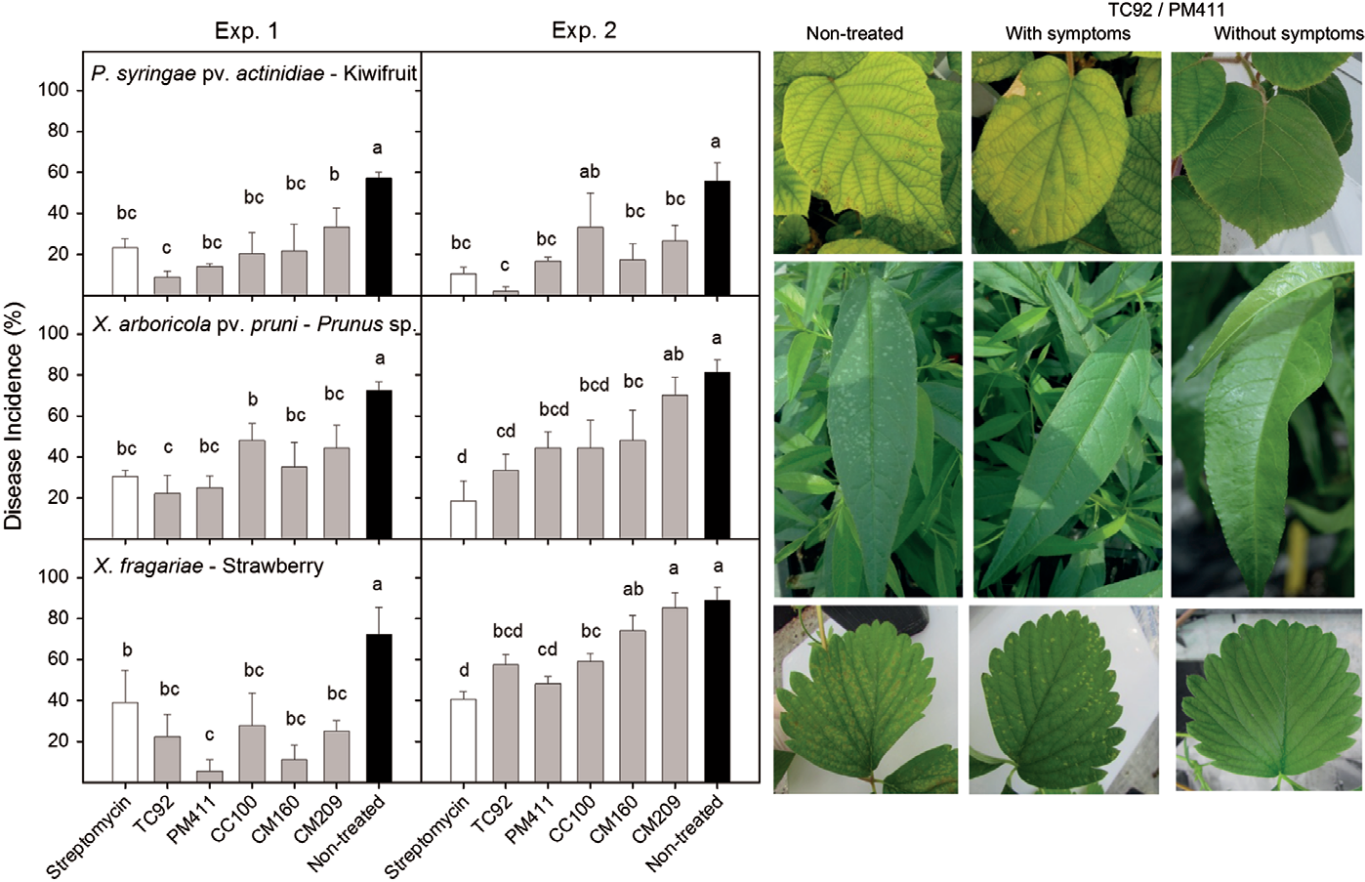
Effect of the treatments with LAB strains (grey bars) on Psa, Xap and *XF* infections in kiwifruit, *Prunus* and strawberry plants, respectively, under greenhouse conditions. The effect of strains on disease incidence (%) was compared with streptomycin (white bars) and a non‐treated control (black bars). Two independent experiments were performed (left and right panels). Values are the mean of three replicates and error bars represent the *SE* of the mean. Bars with the same letter in the same panel do not differ significantly (*p* < 0.05) according to the LSD test. LSD_Psa‐Exp.1_ = 22.4; LSD_Psa‐Exp.2_ = 28.8; LSD_Xap‐Exp.1_ = 23.4; LSD_Xap‐Exp.2_ = 29.0; LSD_Xf‐Exp.1_ = 32.8; LSD_Xf‐Exp.2_ = 16.8. The photographs show the symptoms observed in non‐treated and treated plants. LAB, lactic acid bacteria; LSD, least significant difference; Psa, *Pseudomonas syringae* pv. *actinidiae*; Xap, *Xanthomonas arboricola* pv. *pruni*; *XF*, *Xanthomonas fragariae*

*L. mesenteroides* CM160 and CM209 and *L. plantarum* CC100, PM411 and TC92 were selected for efficacy assays in greenhouse conditions thanks to their broad and high in vitro antagonism against Psa, Xap and Xf. PM411 and TC92 strains consistently reduced Psa, Xap and Xf disease incidence in kiwifruit, *Prunus* and strawberry plants, respectively, in comparison with the non‐treated controls in both experiments performed (Figure 2). The efficacy of the *L. plantarum* strains in kiwifruit ranged from 84.5 to 96.3% for TC92 and from 70.0 to 75.4% for PM411, while in *Prunus* it ranged from 59.1 to 69.3% for TC92 and from 45.5 to 65.5% for PM411 and in strawberry from 35.4 to 69.2% for TC92 and from 45.8 to 92.3% for PM411. PM411 and TC92 did not differ significantly from streptomycin, except for PM411 in one experiment with the Xf‐strawberry pathosystem. CC100, CM160 and CM209, however, did not reduce disease incidence when compared to non‐treated plants in some experiments.

### 3.3 Survival of L. plantarum PM411 and TC92 on leaves

**Figure 3:**
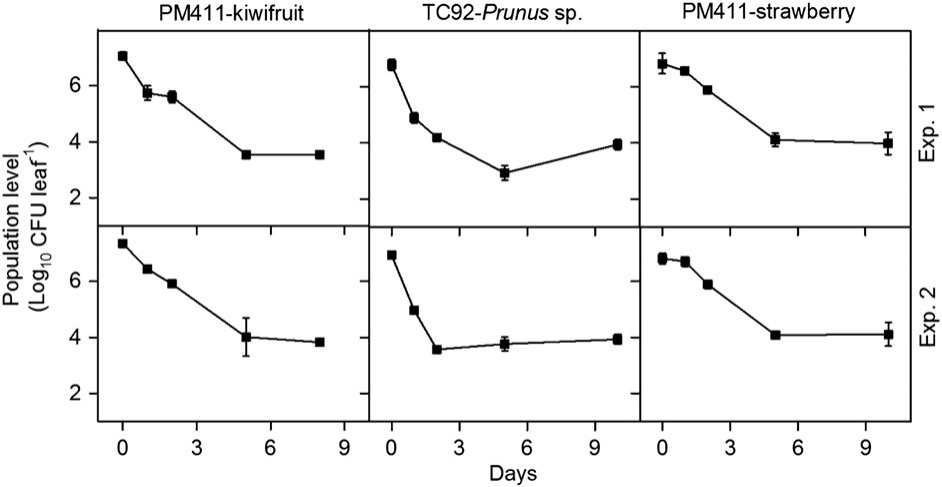
Survival of *Lactobacillus plantarum* PM411 in kiwifruit and strawberry plants and survival of L. plantarum TC92 in *Prunus* plants under controlled environmental conditions. Survival is shown as the population level (Log_10_ CFU leaf^−1^). Values are the mean of the three replicates, and error bars represent the *SE* of the mean. Two independent experiments were performed (top and bottom panels). CFU, colony‐forming units

*L. plantarum* PM411 and TC92 survival on kiwifruit and strawberry plant leaves (PM411) and the leaves of *Prunus* plants (TC92) under greenhouse conditions was monitored (Figure 3). After inoculation, the population level decreased approximately two log units between the 1st and 10th day. In fact, the drop in population was observed during the first 5 days and the viable population remained stable thereafter at around 10^4^ CFU per leaf.

### 3.4 Control of Psa, Xap and Xf in plants in semi‐field and field conditions

**Figure 4:**
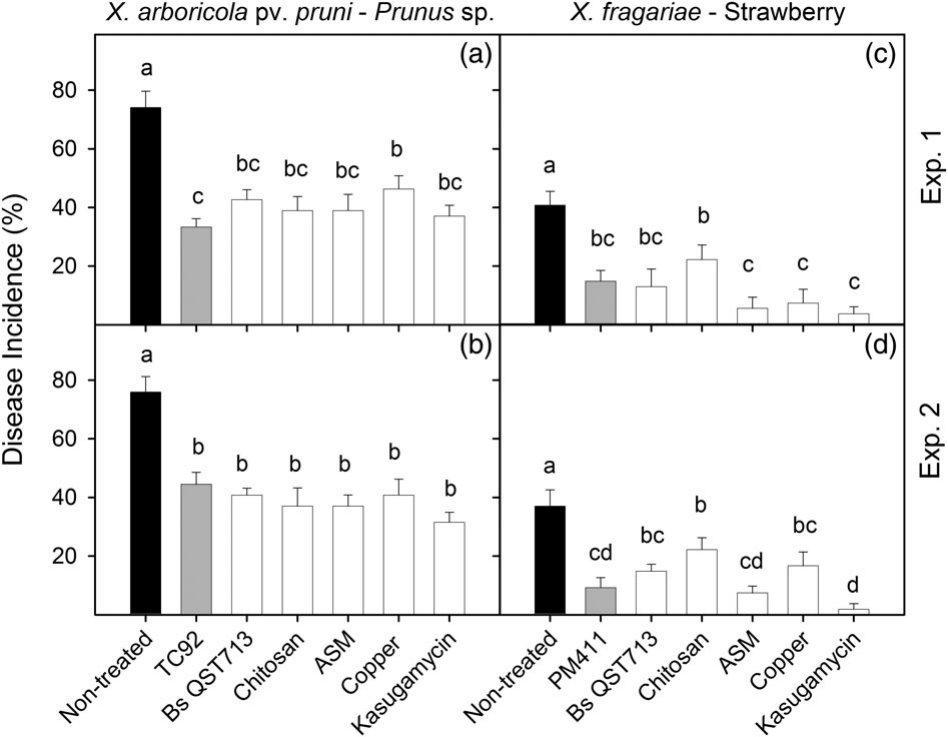
Effect of the treatment with *Lactobacillus plantarum* TC92 and PM411 (grey bars) on Xap infections in potted *Prunus* plants and on Xf infections in potted strawberry plants, respectively, in semi‐field experiments. The effect of strains on disease incidence (%) was compared with different reference products, such as B. subtilis QST713, chitosan, ASM, copper, and kasugamycin (white bars) and a non‐treated control (black bars). Two independent experiments were performed. Values are the mean of three replicates and error bars represent the *SE* of the mean. Bars with the same letter in the same panel do not differ significantly (*p* < 0.05) according to the LSD test. LSD_Xap‐Exp.1_ = 12.7; LSD_Xap‐Exp.2_ = 13.0; LSD_Xf‐Exp.1_ = 12.8; LSD_Xf‐Exp.2_ = 10.6. LSD, least significant difference; Xap, *Xanthomonas arboricola* pv. *pruni*; *Xf*, *Xanthomonas fragariae*

The efficacy of TC92 and PM411 in controlling Xap and Xf, respectively, was compared to reference products in semi‐field experiments (Figure 4). The incidence of infection attained in *Prunus* plants treated with *L. plantarum* TC92 was significantly lower than that of the non‐treated controls in both experiments performed. TC92 efficacy in controlling Xap on *Prunus* (41.5–55%) was not significantly different from *B. subtilis* QST713, chitosan, ASM and kasugamycin in either experiment or from copper in only one experiment. In both experiments, strawberry plants treated with *L. plantarum* PM411 showed a lower incidence of infection than the non‐treated controls did. PM411 efficacy (63.6–75%) in controlling Xf on strawberry did not differ significantly from *B. subtilis* QST713, ASM, copper and kasugamycin in either experiment or from chitosan in only one experiment.

**Figure 5:**
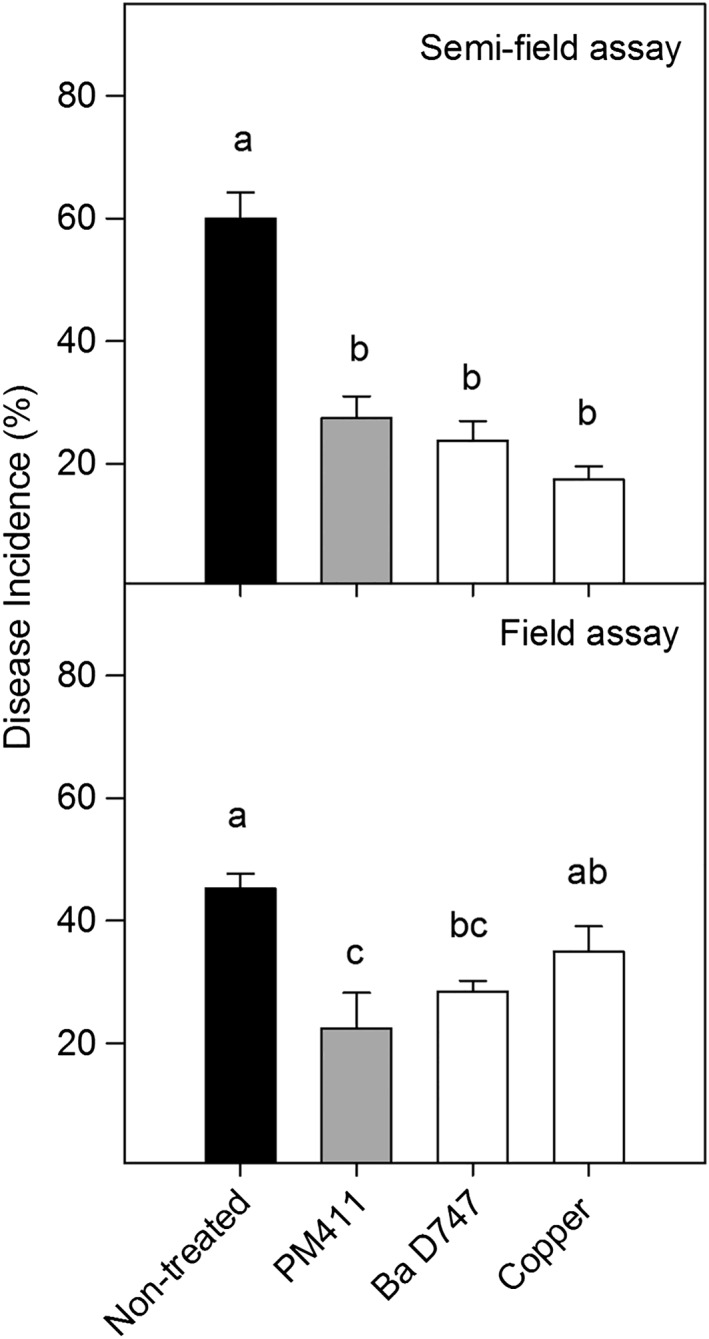
Effect of the treatment with *Lactobacillus plantarum* PM411 (grey bars) on Psa infections in kiwifruit plants in semi‐field and field experiments. The effect of PM411 on disease incidence (%) was compared with reference products such as B. amyloliquefaciens D747 and copper (white bars), and a non‐treated control (black bars). Values are the mean of three replicates and error bars represent the *SE* of the mean. Bars with the same letter in the same panel do not differ significantly (*p* < 0.05) according to the LSD test. LSD_Semi‐field_ = 13.5; LSD_Field_ = 11.7. Ba, B. amyloliquefaciens; LSD, least significant difference; Psa, *Pseudomonas syringae* pv. *actinidiae*

The efficacy of PM411 in controlling Psa was compared to reference products in a semi‐field experiment (Figure 5). PM411 was able to halve disease incidence in kiwifruit plants in comparison to non‐treated controls with significant differences. PM411 efficacy in controlling Psa on kiwifruit (54.2%) was similar to that of *B. amyloliquefaciens* D747 and copper without any significant differences between them.

In the field experiment performed in a commercial kiwifruit orchard, the natural Psa incidence in non‐treated plants was 45.3%. PM411 was the most effective treatment in lowering the incidence of disease, reaching a 50.3% efficacy and no significant differences were observed in comparison with *B. amyloliquefaciens* D747 as a reference product, which showed a 22.7% efficacy (Figure 5). Copper did not significantly reduce disease incidence in comparison to non‐treated controls, showing a 37.1% efficacy.

### 3.5 Psa population suppression by PM411

The epiphytic and endophytic populations of Psa on the leaves of the potted kiwifruit plants treated with PM411 were monitored for 4 days post pathogen inoculation (dpi) in high RH conditions (Table 3). The Psa population recovered from the leaves of the plants treated with PM411 was lower than that of the untreated plants. Specifically, for the epiphytic population, the reduction (1 log unit) was significant at 1 dpi, while for the endophytic population it was observed as being 4 dpi. A reduction in the Psa population from 1.5 to 2 log units was observed in streptomycin‐treated plants.

**Table 3:** Effect of *Lactobacillus plantarum* PM411 treatment on survival of Psa in kiwifruit plants

	Psa population levels^a^							
	Epiphytic (Log_10_ CFU leaf^−1^)				Endophytic (Log_10_ CFU g^−1^)			
Treatment^b^	1 day		4 days		1 day		4 days	
PM411	6.1	b	5.5	ab	3.7	A	2.8	b
Streptomycin	5.4	c	5.0	b	2.7	B	<2^c^	c
Non‐treated	7.2	a	6.5	a	4.0	A	3.6	a
LSD		0.48		1.02		0.58		0.23

CFU: colony‐forming units; LSD: least significant difference; Psa: *Pseudomonas syringae* pv. *actinidiae*.

a Values of Psa population levels are the mean of the three replicates one and 4 days post Psa inoculation on plants. Values with different letters in the same column are significantly different (*p* < 0.05) according to the LSD test.

b Treatments were carried out 1 day before Psa inoculation.

c Log_10_ CFU g^−1^ value under detection limit.

### 3.6 Metabolite and genotypic characterisation of PM411 and TC92

To understand the antibacterial activity of PM411 and TC92, CFS were analysed. While CFS without pH adjustment (pH 3.8) showed antibacterial activity against the three target pathogens (Psa, Xap and Xf), the inhibitory activity was completely lost after CFS pH neutralisation and enzymatic treatments (tripsin, α‐chymotrypsin, proteinase K and catalase) had no impact on the inhibitory effect (data not shown). A similar amount of a mixture of D‐ and L‐lactic acid obtained from both strains was quantified in CFS (75.75 ± 0.63 mM for PM411 and 74.86 ± 1.02 mM for TC92), with D‐lactic acid being the predominant optical isomer in the mixture.

**Figure 6:**
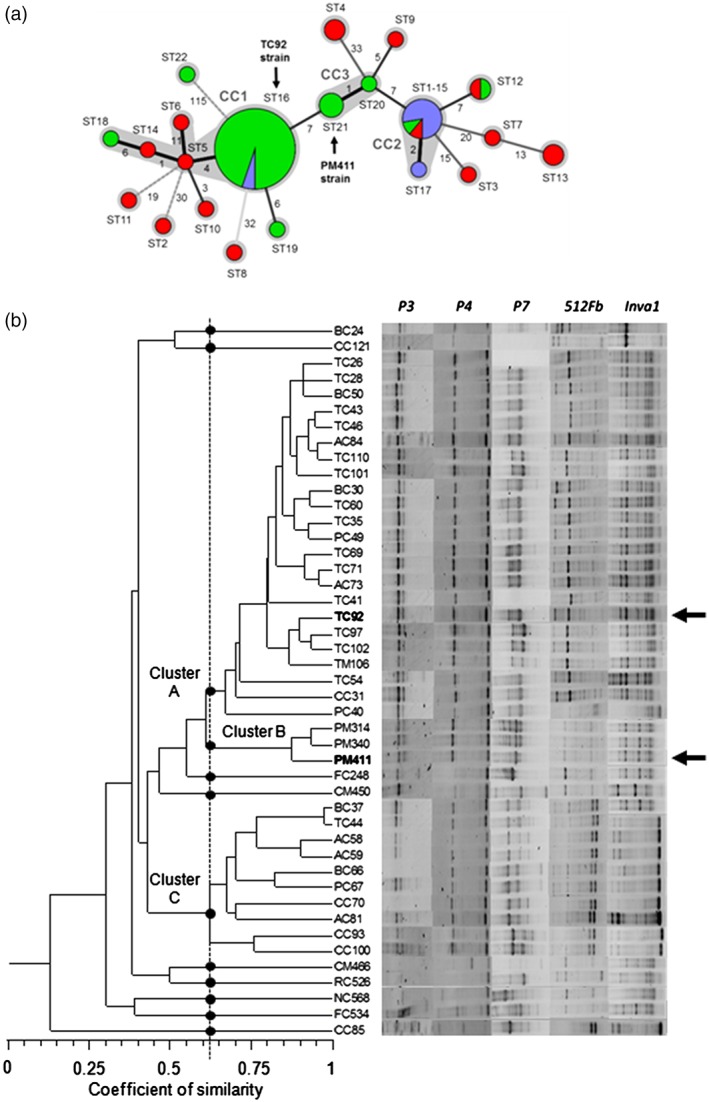
Minimum spanning tree of 71 *Lactobacillus plantarum* isolates based on allelic profiles of the genes *pgm*, *ddl*, *gyrB*, *purK1*, *gdh* and *mutS* (A). Each circle corresponds to an ST, and the size of the circle is proportional to the number of isolates within any given ST. Colour codes of isolates: Green, INTEA‐CIDSAV collection; red, de las Rivas et al. (2006); and blue, Tanganurat et al. (2009). The type of line between isolates indicates the strength of the genetic relationship between them (black, strong relationship; grey, intermediate relationship; and dotted line, weak relationship). The number of mutations between STs is also indicated for each relationship. STs that belong to the same CC are shown as circles grouped in a grey area. Black arrows indicate that the PM411 strain with ST21 belongs to CC3, and the TC92 strain with ST16 belongs to CC1. Dendrogram of the RAPD‐PCR patterns of 45 L. plantarum strains (INTEA‐CIDSAV collection) using primers P3, P4, P7, 512Fb and Inva1 (B). Dots indicate the three main clusters as well as the singletons. Cluster analysis was performed using the (UPGMA), and with the Dice coefficient of similarity. Black arrows indicate TC92 and PM411 strains. CC, clonal complex; INTEA‐CIDSAV, Institute of Food and Agricultural Technology and Center for Innovation and Development of Plant Health; RAPD‐PCR, randomly amplified polymorphic DNA‐PCR; ST, sequence type; UPGMA: unweighted pair group method with the arithmetic average

In relation to genotypic characterisation, the MST analysis of different *L. plantarum* strains based on MLST data (Figure S1, Supporting Information) is shown in Figure 6. Specifically, PM411 and TC92 were analysed together with other isolates of which 43 came from the INTEA‐CIDSAV culture collection and 26 from other studies. The six housekeeping genes yielded a total of 21 STs and the majority of the strains (66%) represented ST16 (*n* = 38) and ST1‐15 (*n* = 9). The 21 STs were grouped into three CCs. CC1 consisted of five STs that accounted for 42 strains, including ST5 as the putative primary founder. CC2 consisted of ST1‐15 represented by nine strains and ST17, which included one strain. CC3 was composed of ST20 and ST21 with four strains. The remaining STs were considered singletons. Specifically, PM411 and TC92 were found in two different CCs: TC92 in CC1 and PM411 in CC3. Strain PM411 shared ST21 with two strains from the INTEA‐CIDSAV collection. Strain TC92 shared ST16 with another 35 strains from the INTEA‐CIDSAV collection and with two strains studied by Tanganurat et al. (2009). The differences between TC92 and PM411 were found in *purK1* and *gdh*.

PM411 and TC92, together with 43 *L. plantarum* strains, were subjected to RAPD‐PCR analysis (Figure 6). According to the band profiles, the strains were divided into three clusters at a coefficient of similarity greater than 0.6. TC92 shared cluster A with 22 strains and PM411 shared cluster B with two other strains.

## 4 DISCUSSION

The growing interest in sustainable management of plant diseases caused by Psa, Xap and Xf has stimulated the search for novel BCA. While several bacteria, such as *Pseudomonas* and *Xanthomonas*, have been selected for studies thanks to their biocontrol activity against these pathogens, the studies in question only focused on a single pathogen (Biondi, Dallai, Brunelli, Bazzi, & Stefani, 2009; Henry, Gebben, Tech, Yip, & Leveau, 2016; Kawaguchi, Inoue, & Inoue, 2014; Wicaksono, Jones, Casonato, Monk, & Ridgway, 2018). In this present work, a multi‐disease approach was taken to select antagonistic bacteria with broad‐spectrum activity. This strategy has been previously used to screen for BCA in other pathosystems (Haidar et al., 2016). As some LAB strains have shown antagonistic activity against bacterial and fungal plant pathogens (Fhoula et al., 2013; Trias, Bañeras, Montesinos, & Badosa, 2008; Visser et al., 1986; Wang et al., 2011) and biocontrol efficacy on plants (Roselló et al., 2013; Roselló et al., 2017; Shrestha, Kim, & Park, 2014; Tsuda et al., 2016), plant‐associated LAB have been considered as candidates for developing BCA.

Five out of 55 LAB strains (CC100, CM160, CM209, PM411 and TC92) were selected as they exhibited a broad spectrum of in vitro antagonism against Psa, Xap and Xf. Other reports have also demonstrated the high antagonistic activity of certain LAB strains against phytopathogenic bacteria such as *Xanthomonas campestris*, *Pectobacterium carotovorum*, *P. syringae*, *Pseudomonas savastanoi* and *Ralstonia solanacearum* (Fhoula et al., 2013; Shrestha et al., 2014; Trias, Bañeras, Montesinos, & Badosa, 2008). in vitro assays are commonly used for the preliminary selection of antagonist strains from a huge number of candidates, testing different target phytopathogens (Kavroulakis et al., 2010; Mora, Cabrefiga, & Montesinos, 2015). This strategy is mainly focused on selecting antagonists, such as LAB (Ben Omar et al., 2008), whose mode of action is the secretion of antimicrobial compounds (Köhl et al., 2011). Therefore, this method allowed BCA candidate strains to be preselected.

*In planta* tests based on a multiple‐pathosystem approach were apt for highlighting strains with potential biocontrol ability and broad‐spectrum activity from among those previously selected in the in vitro tests. The biocontrol activity of PM411 and TC92 against Psa, Xap and Xf was confirmed in plants under greenhouse conditions, as pathogen infections were reduced in the same way as streptomycin. In a previous study, both strains were also selected in a screening procedure because they exhibited a suppressive effect against *Erwinia amylovora* in plant assays (Roselló et al., 2013).

The biocontrol efficacy of PM411 and TC92 against Psa, Xap and Xf in the corresponding host plants was also observed in the semi‐field and field experiments, confirming that they could be useful in a wide range of conditions. Because of the European Union (EU) regulatory quarantine restrictions for pathogens, the assessment of disease management strategies in orchards affected with these pathogens is practically unworkable in designated protected zones such as Spain. Therefore, semi‐field experiments have been performed in this study as they have been previously reported as being comparable to field experiments for biocontrol testing (Cabrefiga, Francés, Montesinos, & Bonaterra, 2011).

Biocontrol efficacy of the selected LAB strains was comparable to *B. amyloliquefaciens* D747, *B. subtilis* QST713, chitosan, ASM, copper and kasugamycin. Because of the restrictions on the use of commercial products, LAB strains could be a promising alternative tool to be included in disease management strategies. In particular, phytotoxicity and pathogen resistance selections, in terms of copper compounds and antibiotics used for Psa, Xap and Xf control, have been reported (Colombi et al., 2017; Lalancette & McFarland, 2007; Roberts, Jones, Chandler, & Stall, 1997). However, with some plant, defence elicitors such as ASM (Reglinski et al., 2013) and commercial microbial biopesticides based on *Bacillus* spp. against Psa (Monchiero, Gullino, Pugliese, Spadaro, & Garibaldi, 2015), a lack of consistency in efficacy has been demonstrated under some limited conditions.

Because of their potential as biological control agents, PM411 and TC92 were characterised in terms of the mechanism involved in the antimicrobial activity against the pathogens as well as genetically typified for their development as BCA. CFSs obtained from cultures of both strains also showed an inhibitory effect, indicating the presence of antimicrobial compounds. In fact, lactic acid production was confirmed in PM411 and TC92 cell cultures. Meanwhile, plantaricin synthesis by both strains is to be expected as a result of the presence of biosynthetic genes (*plnEF*, *plnJK*) with similar levels of expression (Daranas, Badosa, Francés, Montesinos, & Bonaterra, 2018; Roselló et al., 2013). As the pH neutralisation of CFS eliminated the antibacterial effect, acidic pH or the presence of organic acids could account for the main antimicrobial activity. Other studies have reported organic acids as being one of the main mechanisms through which the antimicrobial activity of LAB is exerted against a broad spectrum of target bacteria (Arena et al., 2016). Although in this work plantaricins have not been proven to contribute to Psa, Xap and Xf inhibition, their role should not be dismissed because acidification or acid‐mediated cell membrane disruption may be required to exert an antagonistic effect (Alakomi et al., 2000). Although the role hydrogen peroxide plays in antimicrobial activity was not confirmed in CFS, its production is expected to be favoured on plant surfaces where there are aerobic conditions, and to contribute to pathogen suppression as has been previously reported (Pridmore, Pittet, Praplan, & Cavadini, 2008). It is hypothesized that when *L. plantarum* colonise the plant surfaces the pH will be acidic because of the production of lactic acid or other organic acids resulting from fermentation. Moreover, under aerobic conditions, the formation of hydrogen peroxide could also contribute to the antagonism. Therefore, to fully understand the multifactorial mode of the actions of PM411 and TC92 responsible for disease prevention in plants, further studies are required.

Besides the production of antimicrobial metabolites, the pre‐emptive colonisation of plant tissues susceptible to pathogen infection is an important mechanism underlying BCA effectiveness (Giddens, Houliston, & Mahanty, 2003). As Psa, Xap and Xf enter the host plant through natural openings, such as leaf stomata or wounds, the presence of PM411 and TC92 cells on the leaf surfaces prior to the arrival of pathogens might avoid infection. In particular, preventively spraying PM411 on plants inhibited endophytic and epiphytic Psa populations, indicating a direct effect on the pathogen and in keeping with the reduction in the incidence of disease in the plant experiments. This inhibitory effect that PM411 and TC92 have, was also observed for *E. amylovora* on pear plant surfaces (Roselló et al., 2017). The survival of PM411 and TC92 on kiwifruit, *Prunus* and strawberry plant leaves was confirmed in greenhouse experiments. Similar population levels were reached on the three different plant hosts and were in agreement with their survival previously reported for pear plants (Roselló et al., 2017). Although after spraying a decrease in the population was observed as a result of the harsh conditions on aerial plant surfaces, a constant level of 10^3^ to 10^4^ CFU leaf^−1^ of LAB strains was attained in the 10 days that followed. This decline in population on leaves was also reported for other *L. plantarum* BCA which, interestingly, attained higher population levels at the wounded sites of leaves (Tsuda et al., 2016). Changes in water availability and temperature, nutrient limitation or ultraviolet radiation on leaves can be transiently inadequate for BCA growth (Lindow & Brandl, 2003).

Although the performance of PM411 and TC92 in supressing pathogens is similar and both strains were identified as *L. plantarum*, they were clearly discriminated by the RAPD‐PCR analysis and belong to different CCs according to the MLST. Interestingly, these strains were analysed together with other plant‐associated *L. plantarum* strains and both the RAPD‐PCR and the MLST analysis displayed similar groups.

*L. plantarum* PM411 and TC92 are effective as preventative treatments to control bacterial pathogens representing different genera (*Pseudomonas, Xanthomonas*) and affecting different hosts (kiwifruit, *Prunus*, strawberry). The broad‐spectrum antagonism against bacterial pathogens they exhibited is mainly based on antimicrobial metabolites. Moreover, they inhibited pathogen populations on plant surfaces by suppressing infections. However, population reduction by PM411 and TC92 under nonconductive conditions might compromise plant protection. To achieve the BCA population required for biocontrol, repeated spray applications may be necessary. Therefore, monitoring viable cells could aid the design of a suitable delivery schedule of applications (Daranas et al., 2018). Likewise, the improvement of BCA ecological fitness could be implemented (Daranas, Badosa, et al., 2018). Further studies under different agricultural and climatic conditions are needed to confirm the performance of PM411 and TC92 in the field.

## Supporting information


**Figure S1** Dendrogram according to the multilocus sequence typing (MLST) typing of 71 *Lactobacillus plantarum* strains, including 45 strains from this study (Roselló et al., 2013; Trias, Bañeras, Badosa, & Montesinos, 2008; Trias, Bañeras, Montesinos, & Badosa, 2008) and 26 strains from other studies. The analysis included allelic profiles of the genes *pgm*, *ddl*, *gyrB*, *purK1*, *gdh* and *mutS*. Colour codes of strains: green, this study; red, de las Rivas, Marcobal, and Muñoz (2006); and blue, Tanganurat, Quinquis, Leelawatcharamas, and Bolotin (2009). Cluster analysis was performed by MEGA version 5.1 software (http://www.megasoftware.net) using the unweighted pair group method with arithmetic averages (UPGMA) and the Kimura two‐parameter model (1,000 Bootstrap method). Bootstrap confidence intervals, origin of the isolates, sequence type (ST) and allelic profiles (in brackets) are indicated.
