# Supplementary figures and images for: Biological control of bacterial plant diseases with Lactobacillus plantarum strains selected for their broad‐spectrum activity
Source: Ann Appl Biol. 2018 Nov 26;174(1):92–105. doi: 10.1111/aab.12476 (PMC6334523; doi:10.1111/aab.12476)

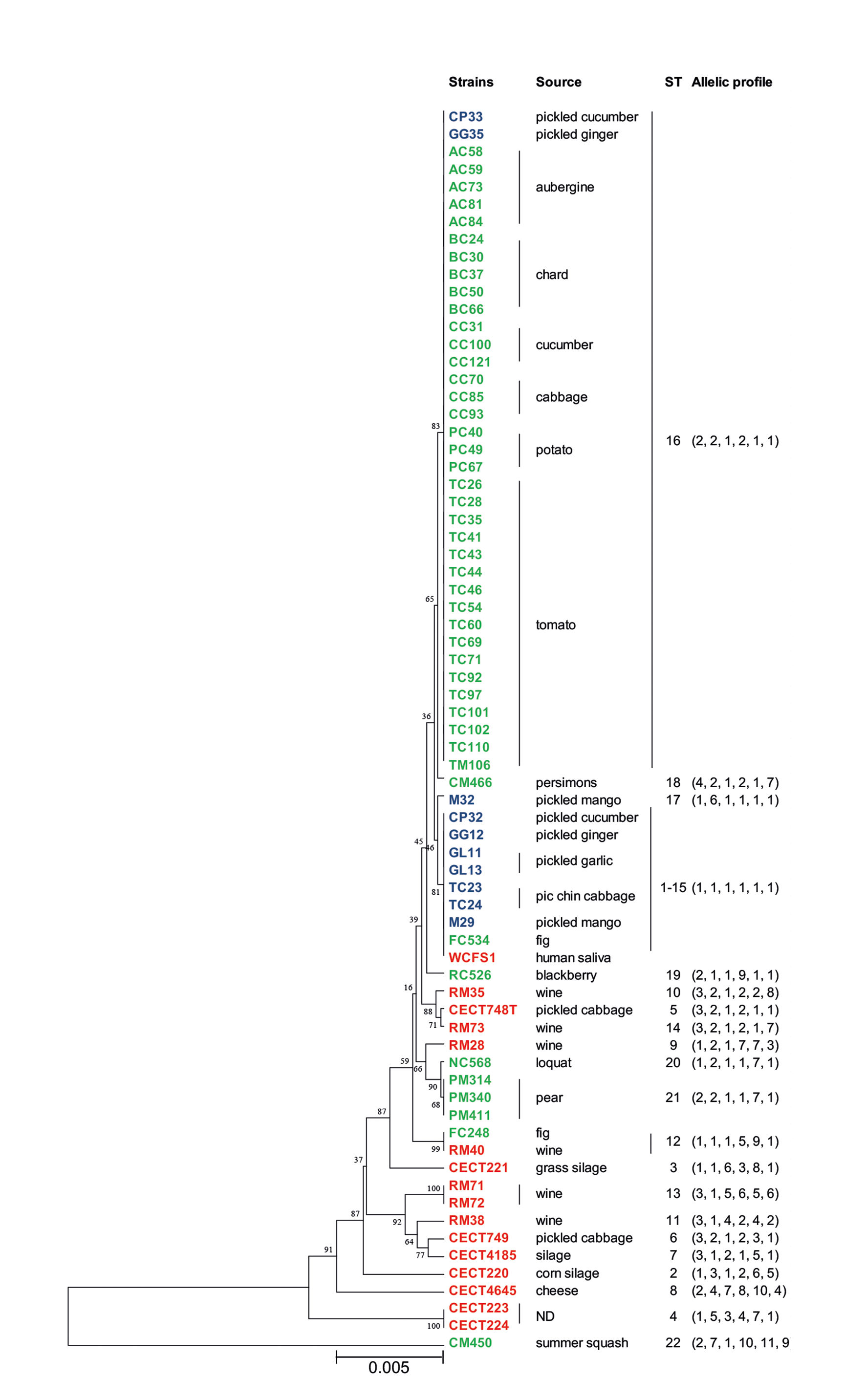

Supplement: Supplementary file 1 — Figure S1 Dendrogram according to the multilocus sequence typing (MLST) typing of 71 Lactobacillus plantarum strains, including 45 strains from this study (Roselló et al., 2013; Trias, Bañeras, Badosa, & Montesinos, 2008; Trias, Bañeras, Montesinos, & Badosa, 2008) and 26 strains from other studies. The analysis included allelic profiles of the genes pgm, ddl, gyrB, purK1, gdh and mutS. Colour codes of strains: green, this study; red, de las Rivas, Marcobal, and Muñoz (2006); and blue, Tanganurat, Quinquis, Leelawatcharamas, and Bolotin (2009). Cluster analysis was performed by MEGA version 5.1 software (http://www.megasoftware.net) using the unweighted pair group method with arithmetic averages (UPGMA) and the Kimura two‐parameter model (1,000 Bootstrap method). Bootstrap confidence intervals, origin of the isolates, sequence type (ST) and allelic profiles (in brackets) are indicated. [file AAB-174-92-s001.tif]
